# Evaluating the nanotoxicity and antioxidant pathway modulation of MnO_2_ nanoparticles in zebrafish

**DOI:** 10.1038/s41598-026-49316-2

**Published:** 2026-05-04

**Authors:** Hanny Tika Draviana, Istikhori Fitriannisa, Sadang Husain, Yu-Tai Chiou, Tsung-Rong Kuo, Chih-Yu Chen

**Affiliations:** 1 International PhD Program in Biomedical Engineering, College of Biomedical Engineering, Taipei Medical University, Taipei City, 110 Taiwan; 2 Department of Physics, Faculty of Mathematics and Natural Science, Lambung Mangkurat University, Banjarmasin, 70124 Indonesia; 3 Graduate Institute of Biomedical Materials and Tissue Engineering, College of Biomedical Engineering, Taipei Medical University, Taipei City, 110 Taiwan; 4 Graduate Institute of Nanomedicine and Medical Engineering, College of Biomedical Engineering, Taipei Medical University, Taipei City110, Taiwan; 5 Department of Orthopedics, Shuang Ho Hospital, Taipei Medical University, Zhongzheng Road, Zhonghe District, New Taipei City, 235 Taiwan; 6 School of Biomedical Engineering, College of Biomedical Engineering, Taipei Medical University, Taipei City, 110 Taiwan

**Keywords:** MnO_2_ nanoparticle, Reactive oxygen species, Toxicity, Oxidative stress, Biochemistry, Biological techniques, Biotechnology, Environmental sciences, Nanoscience and technology

## Abstract

Metal oxides-particularly MnO_2_ nanoparticles (NPs)-have shown great promise due to their unique physicochemical properties, such as biocompatibility, catalytic activity, stimulus-responsiveness, Fenton-like reactivity, and enzyme-mimicking behavior. Nonetheless, applying MnO_2_ NPs in vivo continues to raise concerns about their toxicity and biological impacts on organisms. In this study, MnO_2_ NPs were synthesized via a simple one-pot method by directly mixing potassium permanganate (KMnO_4_) with poly(allylamine hydrochloride) until complete conversion was achieved, followed by characterization of their physical and chemical properties. The toxicity of MnO_2_ NPs was observed by analyzing morphological changes, mortality rates, and gene expressions. The morphological development of zebrafish after being treated with MnO_2_ NPs at concentrations of ≤ 0.1 mg/mL indicated that the material was largely biocompatible with early zebrafish embryogenesis. Mortality data demonstrated that a low concentration (0.025 mg/mL) of MnO_2_ NPs was relatively safe, while higher doses (≤ 0.1 mg/mL) may impair survival due to cumulative oxidative stress or related nanotoxicity effects. Meanwhile, a qPCR analysis revealed that at high MnO_2_ NP concentrations, expressions of *sod1* and *sod2* increased, but *cat* and *gpx1* expressions remained relatively stable, indicating that MnO_2_ NPs act directly as an H_2_O_2_ detoxification agent and maintain cell homeostasis without causing zebrafish death at low concentrations.

## Introduction

Over the past few years, advancements in nanotechnology have actively contributed to shaping global economic growth across various industrial sectors, including biotechnology, medicine, electronics, cosmetics, materials science, and agriculture^1–3^. Various wide-ranging types of nanoscale materials are continuously being developed at a rapid rate for academic and industrial purposes. Among these, nanoparticles (NPs) have become one of the most widely used materials due to rapid advancements in nanotechnology^4–6^. NPs have rapidly been developed for biomedical applications, encompassing diverse diagnostic approaches that utilize advanced imaging technologies, as well as therapeutic uses such as targeted drug delivery, tissue regeneration, orthopedic prosthetics, implant development, and gene and stem cell therapy. In addition, NPs have been extensively investigated for antiviral purposes, for addressing antibiotic resistance, and for advancing vaccine and immunization development^7^. Among various types of NPs, metal oxide NPs stand out due to their unique physicochemical properties, making them particularly valuable in these biomedical fields. The unique physicochemical properties of metal oxides include biocompatibility, chemical stability, and catalytic, optical, magnetic, and electrical properties arising from their nano size and shapes^8–10^. Metal oxide NPs, especially MnO_2_NPs, have become the main focus in various science and technology studies because they have properties such as tunable morphologies, stimulus-responsiveness, a strong oxidizing ability, Fenton-like reactivity, fluorescence resonance energy transfer (FRET) properties, and unique enzyme-mimicking activities^11–13^. MnO_2_ NPs exhibit antitumor and antibacterial properties due to their ability to generate hydroxyl radicals (⋅OH) via Fenton-like reaction between Mn^4+^ and H_2_O_2_. In addition, Mn^2+^ can activate the stimulation of interferon pathway gene (STING) pathways and enhance immune responses, which makes it able to act as a nano-adjuvant. As a carrier, MnO_2_ NPs can increase the water solubility of insoluble drugs. In addition, MnO_2_NPs can also reduce oxidative stress and inflammatory responses, making them widely applicable in wound healing, spinal cord injury repair, and treatment for skin and nervous system disorders^14–17^.

The advantage of MnO_2_ NPs is their ability to generate reactive oxygen species (ROS), which elevate oxidative stress and induce cancer cell death, which is beneficial in cancer therapy. Interestingly, MnO_2_NPs also have the ability to reduce oxidative stress and inflammatory responses under certain conditions due to their catalytic activity^18^. The toxicity effect of MnO_2_NPs also needs to be considered. ROS production and oxidative stress are generally the most common indicators when observing NP toxicity^19^. Oxidative stress refers to a physiological state characterized by an imbalance between the generation and buildup of ROS within cells and tissues and the capacity of biological systems to neutralize or eliminate these reactive molecules^20^. In the process of oxidative stress, the production of reactive species, e.g., HOCl, ONOO⁻, and ⋅OH, directly oxidizes macromolecules, including enzymes, structural proteins, lipids, and nucleic acids, causing cells to function abnormally and die. Oxidative stress has been implicated in the development of multiple disorders, such as Alzheimer’s disease, atherosclerosis, chronic obstructive pulmonary disease, cardiovascular complications, diabetes, psychiatric diseases, renal diseases, and cancers, indicating that various mechanisms enable oxidants to induce cellular damage^21–23^. Understanding the mechanism of NP toxicity and its effects on biological conditions is very important for developing safer NPs for medical applications^24^.

Numerous studies reported the application of MnO_2_ NPs and their toxicity in various biological systems. Z. Wang et al. successfully synthesized MnO_2_@PtCo nanoflowers that triggered biochemical reactions in cells to produce free radicals as tumor therapy by utilizing PtCo to decompose H_2_O_2_ and MnO_2_to increase the production of cytotoxic -OH radicals, thereby causing significant oxidative damage to cancer cells both under normal and low-oxygen conditions without external stimulation^25^. J.Y. Choi et al. found that incubation of MnO_2_in human (A549) lung adenocarcinoma cells for 2 days significantly increased oxidative stress, although the mechanism of toxicity was not clear^26^. Then L. Lin et al. developed mSiO_2_-DOX (doxorubicin)-MnO_2_ (MS@MnO_2_) as a chemotherapeutic agent and chemodynamic therapy (CDT) enhanced by glutathione (GSH) depletion, indicating that MnO_2_ can be a chemodynamic agent that produces cytotoxic -OH with higher effectiveness than Mn^2+^-based sonodynamic therapy (SDT)^27^. A.A. Hafez et al. demonstrated that MnO_2_-NPs significantly influenced mitochondrial function by promoting ROS generation, which subsequently suppressed the activity of mitochondrial complexes II and IV. This disruption contributed to a diminished antioxidant capacity and may trigger pathological processes such as carcinogenesis, apoptosis, and neurodegenerative diseases^28^. M. Li et al. proposed the F127@CNs-CuS/MnO_2_ nano platform, and results showed that in addition to producing oxygen to overcome hypoxia in the tumor environment through reaction with H_2_O_2_in cancer cells, this material also interacted with overexpressed GSH, thereby increasing the effectiveness of combination therapy^29^..

Utilization of MnO_2_ NPs in biological systems requires a thorough understanding of their toxicity profile and biological responses they generate. The zebrafish (*Danio rerio*) organism model is a popular model for assessing toxicity effects of NPs due to its genetic similarity to humans (~ 70%), optical transparency which facilitates observations of embryonic development, representative biological responses, high embryo production, a relatively short life cycle with rapid development, drug responses similar to humans, and low and practical maintenance costs^30–32^. This model enables direct observation of morphological changes, mortality rates, and gene expressions following exposure to a material. Herein, we investigated the effects of MnO₂ NPs on zebrafish embryo development. The properties of MnO_2_ NPs were confirmed through characterization using Raman spectroscopy, ultraviolet-visible (UV-Vis) spectroscopy, scanning electron microscopy (SEM), transmission electron microscopy (TEM), high-resolution (HR)-TEM, and energy dispersive x-ray (EDX) spectroscopy. The development activity of zebrafish in response to MnO_2_ NPs was examined by analyzing morphological changes, mortality rates, and regulation of gene expressions related to oxidative stress and apoptosis using quantitative polymerase chain reaction (qPCR) techniques. Results of this study are expected to provide a comprehensive picture of the effects of MnO_2_ NPs on biological systems and their implications in the biomedical field.

## Materials & methods

### Ethical approval

All experiments in this study were conducted in accordance with the “Guide for the Care and Use of Laboratory Animals” (Eighth Edition, 2011. ILARCLS, National Research Council, Washington, D.C.) and were approved by the Animal Care and Utilization Committee of Taipei Medical University (Approval No. LAC2025-0173). All methods were performed in accordance with the ARRIVE guidelines (https://arriveguidelines.org). Euthanasia of live zebrafish was performed by submersion in ice water (5 parts ice/1 part water, 0–4 °C) for at least 10 min until cessation of opercular movement.

## Materials

Poly(allylamine hydrochloride) (PAH) was purchased from Sigma-Aldrich (St. Louis, MO, USA). Ethanol dehydrate, NaCl, CaSO_4_, KH_2_PO_4_, MgSO_4_, K_2_HPO_4_, and TRIzol reagent were purchased from Bioman Scientific (Taipei, Taiwan). Potassium permanganate (KMnO_4_) was purchased from Acros Organic™ (Thermo Scientific™, Taipei, Taiwan). The TOOLSQuant II Fast RT Kit was purchased by Biotools (New Taipei City, Taiwan). A primer and a reference gene (*rpl13a*) were supplied by the Genomics Research Center, Academia Sinica (Taipei, Taiwan). A LightCycler^®^ 480 System was purchased from Roche (Taipei, Taiwan).

### Synthesis of MnO_2_ NPs

MnO_2_ NPs were synthesized using a one-pot method. In the initial stage, 77 mg of PAH was dissolved in 2 mL of deionized water (DIW). Separately, 66.4 mg of potassium permanganate (KMnO_4_) was also dissolved in 18 mL of DIW until completely dissolved. Next, the PAH solution was slowly added to the KMnO_4_ solution while stirring gently at room temperature for 15 min. Afterward, the solution containing MnO_2_ nanoparticles was purified through centrifugation and washing. To determine the MnO_2_ NP concentration, 1 mL of the reaction mixture was transferred to a vial, dried overnight, and then weighed the next day. The Mn concentration of MnO_2_ NP was also measured by inductively coupled plasma mass spectrometry (ICP-MS). Furthermore, MnO_2_ samples were subjected to characterization tests to determine their physicochemical properties. A UV-Vis spectrometer (Jasco-V770, Sunway Scientific, Tokyo, Japan) was used to determine the absorbance of the materials. An x-ray diffraction (XRD) spectrometer (Bruker, Billerica, MA, USA), operating at 30 mA and 30 kV with Cu Kα radiation, was used to measure the XRD pattern of MnO_2_ NPs. A Raman spectrometer (UniDRON Laser Spectroscopy Confocal Micro Raman Spectrometer, CLT, New Taipei City, Taiwan) was used to analyze the molecular structure, chemical bonds, and vibrations of the samples. TEM (HT-700, Hitachi, Tokyo, Japan) and HR-TEM (JEM-2100 F, JEOL, Tokyo, Japan) were used to evaluate the NPs’ internal structure. Morphological features of the material’s surface were examined and visualized using SEM (SU-3500, Hitachi) that was fitted with an EDX spectrometer (Quantax EDX, Bruker).

### Experimental animals

Adult zebrafish (8–12 months old) were sourced from the Zebrafish Core Laboratory at Taipei Medical University and maintained in a circulating water system (90 × 60 × 45 cm) at 28 °C under a 14-h light and 10-h dark photoperiod. Fish were fed pellet food twice daily. Adult zebrafish naturally spawned between 09:00 and 10:00 each day, after which fertilized eggs were collected and transferred to freshly prepared artificial freshwater (AFW) for incubation. The AFW consisted of 0.2 mM CaSO_4_, 0.2 mM MgSO_4_, 0.5 mM NaCl, 0.16 mM KH_2_PO_4_, and 0.16 mM K₂HPO₄ (Sigma-Aldrich, St. Louis, MO, USA), with the pH adjusted to 6.8–7.0.8.0. Embryos up to 5 days post-fertilization (dpf) were used in this study. For the toxicity analysis of NPs, 10 embryos were combined per test, and each test was repeated 10 times. The exposure volume was 1 mL, with the exposure medium renewed every 24 h. MnO_2_ NPs were tested at four concentrations, including control, 0.025, 0.05, and 0.1 mg/mL. The acceptance criteria followed OECD Test Guideline 236.

### Messenger (m)RNA extraction and a qPCR

For mRNA extraction and the qPCR analysis, we combined 10 embryos into one sample. During each experiment, 10 embryos were collected as one sample (*n* = 10) from experimental groups of the same concentration. For each concentration in this study, we took five sets for averaging (*n* = 5). Materials were divided into four concentrations (control, and 0.025, 0.05, and 0.1 mg/mL) for the qPCR analysis. Total RNA was extracted from zebrafish embryos using the TRIzol reagent following the manufacturer’s protocol. The isolated RNA was subsequently reverse-transcribed into complementary (c)DNA using a TOOLSQuant II Fast RT Kit. A real-time qPCR was performed on a LightCycler 480 system, using a final reaction volume of 10 µL, containing cDNA at a concentration of ≥ 5 ng/µL. Each reaction mixture consisted of cDNA, 50 nM primers, and LightCycler^®^480 SYBR Green I Master^33^. Expression levels of target genes were normalized to the *rpl13a* reference gene. qPCR amplification was carried out under the following conditions: initial denaturation at 95 °C for 10 min, followed by 35 cycles of denaturation at 95 °C for 10 s, annealing at 60 °C for 20 s, and extension at 72 °C for 20 s. A melting curve analysis was performed at 95 °C for 15 s, followed by 60 °C for 1 min, and then 95 °C for 15 s at the end of the amplification process. The relative mRNA expression was calculated using the comparative Ct (ΔΔCt) method, with *rpl13a* serving as the internal control (housekeeping) gene.

## Results

### Characterization of MnO_2_ NPs

**Fig. 1:**
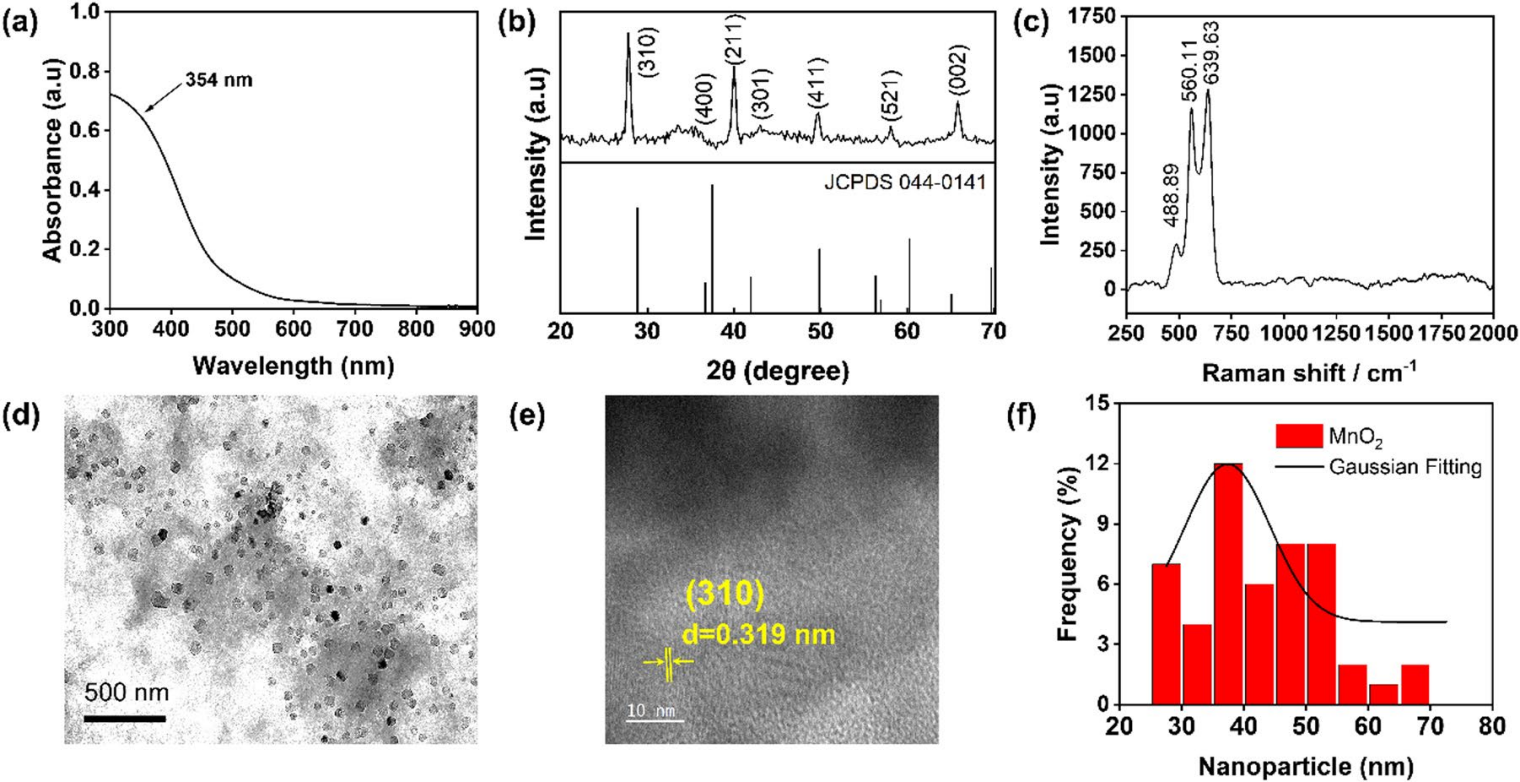
(**a**) Absorption spectra of MnO_2_, (**b**) XRD pattern of MnO_2_, (**c**) Raman spectra of MnO_2_, (**d**) TEM images of MnO_2_, (**e**) HR-TEM of MnO_2_, and (**f**) size distribution of MnO_2_ using Gaussian fitting.

MnO_2_ NPs were synthesized through the direct combination of aqueous solutions containing KMnO_4_ and PAH (15 kDa). The composition and morphology of MnO_2_ NPs were identified using UV-Vis, XRD, Raman spectroscopy, TEM, HR-TEM, SEM, and EDX analyses. To analyze the optical properties of MnO_2_ NPs, analyses were conducted to determine the absorption spectra using UV-Vis spectroscopy. As shown in Fig. 1a, the UV-Vis spectrum of MnO_2_NPs exhibited the highest peak at a wavelength of 354 nm. This behavior can be attributed to the photon-induced excitation of electrons from the valence band to the conduction band^34^. Furthermore, to determine the crystalline structure of MnO_2_, its composites, and impurities, an XRD analysis was performed. Figure 1b presents the XRD pattern of MnO_2_ NPs, which exhibits strong agreement with the standard α-MnO_2_ phase (JCPDS no. 044–0141). Characteristic diffraction peaks appearing at 2θ values of 27.79, 35.52, 40.0, 43.05, 49.77, 58.11, and 65.84 respectively corresponded to the (310), (400), (211), (301), (411), (521), and (002) crystallographic planes. The distinct and sharp diffraction peaks observed confirmed the highly crystalline nature of the synthesized MnO_2_NPs^35^. Local structural features and phase composition of MnO_2_ NPs were further characterized using Raman spectroscopy, as illustrated in Fig. 1c. The spectrum reveals three distinct Raman bands positioned at 488.89, 560.11, and 639.63 cm^− 1^. The prominent band at 639.63 cm^− 1^ corresponded to the A_1g_ symmetric stretching mode associated with the $\:{C}_{2h}^{3}$ space group, representing v2 (Mn-O) vibrations within MnO_6_ octahedral units. Meanwhile, the band appearing at 560.11 cm^− 1^ was assigned to v3 (Mn-O) stretching vibrations occurring in the basal plane of [MnO_6_] layers. The lower-frequency band centered at 488.89 cm^− 1^ was attributed to Mn-O-Mn bending vibrations within the MnO_2_octahedral framework^36,37^. Further characterization was performed to investigate topographical features and the average particle size of MnO_2_ NPs using TEM. Based on Fig. 1 d, MnO_2_ NPs had an average size of about 40 nm. Furthermore, HR-TEM images were analyzed to confirm the structural information of MnO_2_ and ascertain whether MnO_2_ NPs indeed possessed two types of crystallinity. As illustrated in Fig. 1e, MnO_2_ NPs displayed a lattice fringe pattern measuring 0.32 nm, corresponding to the (310) crystal plane as observed, in agreement with the JCPDS no.044–0141 reference. The particle size of MnO_2_ NPs was further validated using a Gaussian fitting curve simulation. As observed in Fig. 1f, this evidence is presented based on a histogram graph displaying the size distribution of MnO_2_ NPs based on data from 50 NPs presented in a TEM image. Based on the Gaussian fitting curve simulation, it was determined that the average particle size of MnO_2_ NPs was 37.4 ± 3.35 nm.

**Fig. 2:**
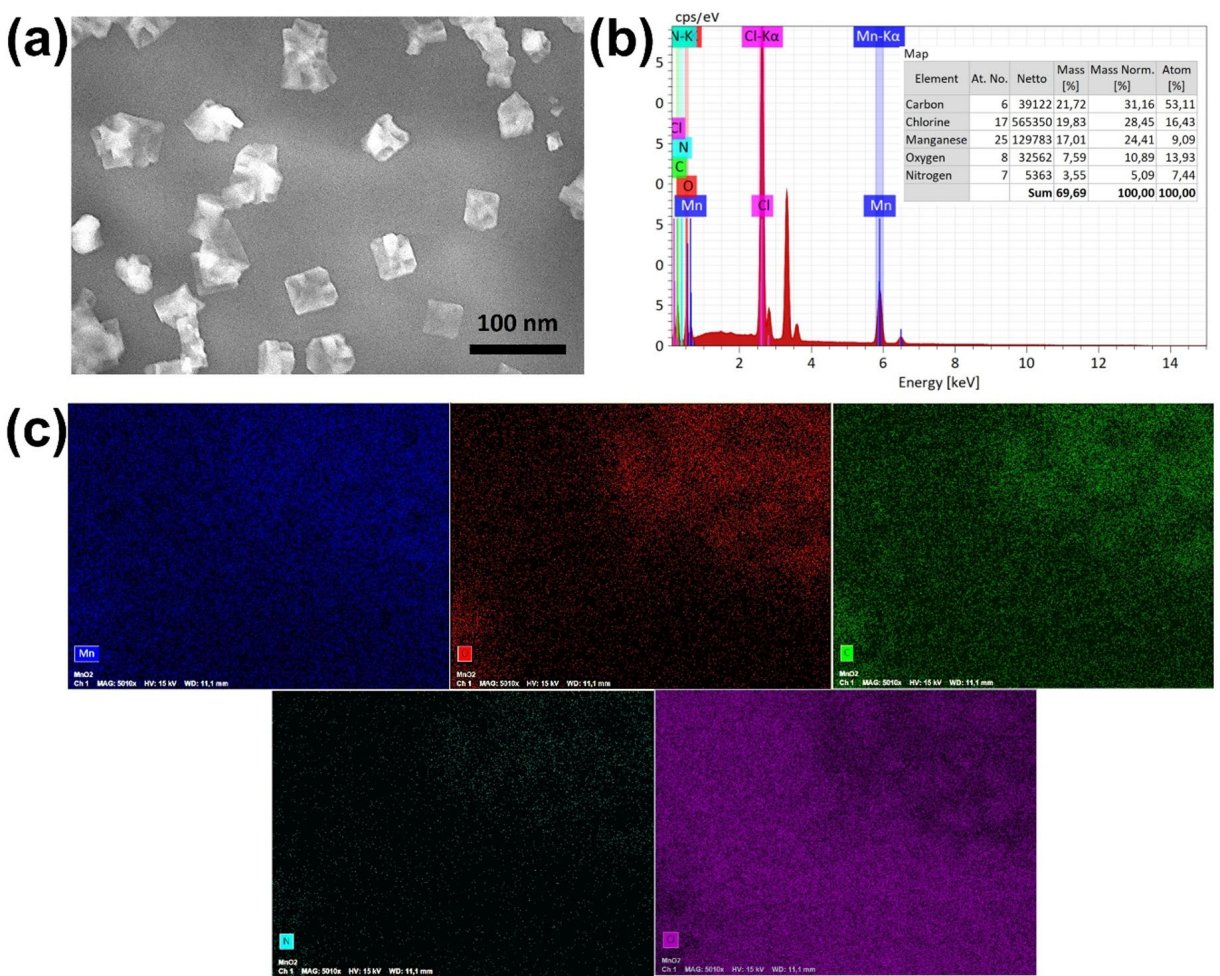
(**a**) SEM images, (**b**) EDX spectrum, and (**c**) elemental mapping of MnO_2_ NPs.

MnO_2_ NPs were analyzed using SEM to gain a deeper understanding of their morphology and surface properties. As seen in Fig. 2a, the crystal structure of MnO_2_ exhibited a cubical shape surface. SEM images were further utilized to determine the EDX elemental distribution. As depicted in Fig. 2b, MnO_2_ NPs comprised 31.16% carbon, 28.45% chlorine, 24.41% manganese, 10.89% oxygen, and 5.09% nitrogen. The elemental distribution maps of each element on MnO_2_ NPs are also shown in Fig. 2c. Overall, based on the characterizations conducted, including UV-Vis, Raman spectroscopy, XRD, TEM, HR-TEM, SEM, and EDX analyses, it was concluded that the synthesis of MnO_2_ NPs using the one-pot system method was successful.

### Morphological development of zebrafish at 24, 48, and 72 hpf

**Fig. 3:**
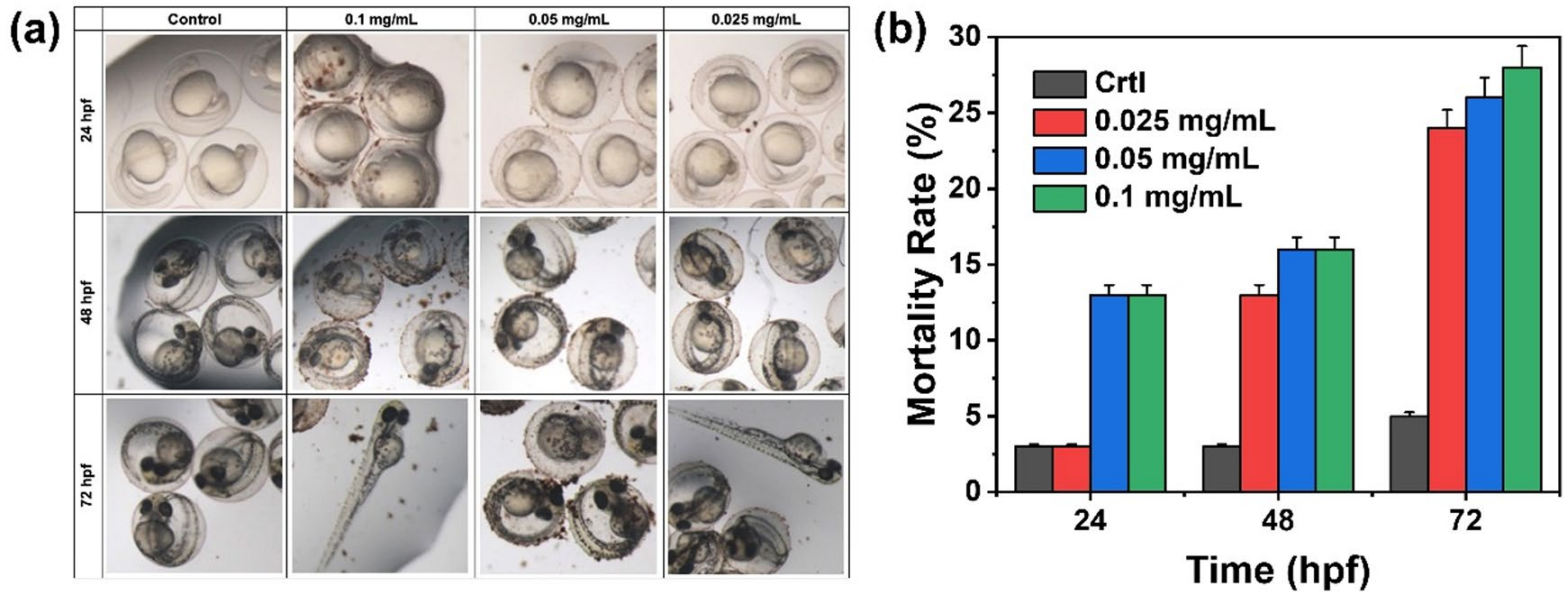
(**a**) Morphology of developing zebrafish embryos, and (**b**) mortality rates of zebrafish embryos, treated with 0.025, 0.05, and 0.1 mg/mL MnO_2_ NPs at 24, 48, and 72 hpf.

To evaluate the biosafety of the NPs, the Fish Embryo Acute Toxicity (FET) test was performed following OECD Test Guideline 236. Zebrafish (*D. rerio*) embryos were utilized as a robust in vivo model due to their significant genetic similarity to humans and physiological relevance^38–40^. Zebrafish exhibit rapid growth, reaching maturity within approximately 3 months, and their larvae develop fully functional organs within only 5 days post-fertilization (dpf). Furthermore, zebrafish embryos are optically transparent, enabling real-time monitoring of organogenesis and allowing the direct observation of malformations caused by NP exposure. In this study, the toxicity of MnO_2_NPs was assessed by examining the morphological development of zebrafish embryos at three critical developmental stages-24, 48, and 72 h post-fertilization (hpf)-following exposure to three MnO₂ NP concentrations (0.025, 0.05, and 0.1 mg/mL), which correspond to Mn concentrations of 0.00725, 0.0145, and 0.029 mg/mL, respectively. As demonstrated in Fig. 3a, at 24 hpf, embryos across all treatment groups, including the controls, showed normal early developmental patterns. Specifically, principal embryonic structures such as segmented somites, otoliths, the developing optic primordium, the elongating tail or notochord, and the initial yolk sac extension could clearly be observed^41^. These observations indicated no significant developmental disruption at early embryonic stages due to NP exposure. Additionally, embryos displayed typical spontaneous tail movements, a characteristic of normal embryonic viability at this stage. Notably, even embryos subjected to the highest NP concentration presented comparable morphological features and developmental timing to those of control embryos, without detectable delays in body axis formation or segmentation. At 48 hpf, the uptake of MnO_2_ NPs by zebrafish embryos was observed. At this stage, organogenesis of zebrafish embryos had significantly progressed across all treatment groups. Major sensory organs, including pigmented eyes and auditory structures, were well-formed, alongside distinct brain regions, such as the optic tectum, and defined craniofacial features. Functional musculature and segmented somites, neural structures including the spinal cord, and initial development of fins (caudal and pectoral fins) were also apparent. Furthermore, prominent melanocyte pigmentation patterns emerged on the head and trunk regions. Importantly, no malformations such as spinal curvature, edema, or growth impairments were observed in embryos exposed to MnO_2_ NPs, suggesting that NP exposure at these concentrations did not impede typical embryonic development timelines. By 72 hpf, zebrafish embryos had achieved substantial anatomical maturity and were preparing for or undergoing hatching. Larvae across all concentrations, including the highest MnO_2_ NP exposure (0.1 mg/mL), displayed indistinguishable morphological features compared to the control group, including a straight body axis, fully formed notochord, robust blood circulation, and a beating heart. Critical cardiovascular structures such as aortic arches, dorsal aorta, and pericardium were fully developed. Additionally, inflated swim bladders, protruding pectoral fins, and well-differentiated eyes, auditory organs, and mouth structures were consistently observed. Embryos surviving to 72 hpf maintained intact morphological features, including dorsal and ventral fin folds, somite-derived musculature, and a well-defined neurocoel. These comprehensive observations strongly indicated that MnO_2_NPs, at concentrations of 0.025–0.1.025.1 mg/mL, did not significantly disrupt anatomical development through 3 dpf. During zebrafish embryogenesis, the yolk serves as a primary nutritional reserve, composed largely of endogenous lipids stored in yolk globules. Yolk sacs can also act as reservoirs for absorbed chemicals, which can potentially induce edema and other abnormalities under toxic conditions^42^. However, throughout this study’s observational period, no yolk sac edema was detected in embryos exposed to MnO_2_ NPs. Similarly, there was an absence of morphological abnormalities such as spinal curvature, tail deformation, or dwarfism. Overall, our findings reinforce that in zebrafish embryos exposed to MnO_2_ NPs, within the tested concentration range (0.025–0.1.025.1 mg/mL), gross morphological integrity and normal timing of developmental milestones indicated considerable biocompatibility.

### Mortality and dose-dependent toxicity

Mortality of zebrafish embryos (24–72 hpf) exposed to MnO_2_ NPs at different concentrations, as presented in Fig. 3b, shows a clear dose- and time-dependent increase in mortality (error bars denote ±standard deviations (SDs)). As development progressed, higher NP doses led to greater embryo lethality. At 24 hpf, all groups showed high survival (mortality ranged from 3% to 13% even at 0.1 mg/mL), but by 48 hpf divergence emerged: embryos exposed to 0.05 and 0.1 mg/mL MnO_2_ had notably higher cumulative mortality (16%) than the controls (3%) and those exposed to 0.025 mg/mL (13%). By 72 hpf, mortality had further increased in a concentration-dependent manner, reaching approximately 28% in the 0.1 mg/mL group, 26% at 0.05 mg/mL and 24% at 0.025 mg/mL, versus only 5% in the controls. This trend of increasing mortality with both NP concentration and exposure duration indicated that while low doses of MnO_2_NPs were largely tolerated, higher doses gradually exceeded the embryos’ capacity to cope, leading to death in a fraction of the population. Such dose- and time-dependent toxicity is a common observation in nanomaterial studies. For example, chronic exposure to high levels of SeNPs and ZnONPs caused significantly elevated zebrafish embryo mortality relative to low-dose exposures^43^. In our study, despite substantial mortality at elevated NP concentrations, approximately 73% of embryos survived at the highest NP dose (0.1 mg/mL), indicating primarily sublethal stress rather than acute toxicity (e.g., slight hatching delays or reduced sizes), even though overt malformations were not observed. This was proven and supported by microscopic morphological observations. Overall, the mortality data demonstrated a clear threshold-dependent effect of MnO_2_NPs: mortality at 0.025 mg/mL, moderate mortality at 0.05 mg/mL, and significant mortality at 0.1 mg/mL by 72 hpf. This aligns with the general nanotoxicology paradigm that NP toxicity intensifies with an increasing dose and exposure time^43^. It also underlines that while low concentrations of MnO_2_ NPs appear relatively safe for early development, higher concentrations (≥ 0.1 mg/mL in this case) can compromise survival, likely through cumulative oxidative stress or other nanomaterial-induced insults over time.

### Mechanisms of synergistic effects of MnO_2_ NPs to zebrafish embryo growth

Expressions of antioxidant defense genes were analyzed to investigate how zebrafish embryos responded to exposure to MnO_2_ NPs. We examined relative expression levels of *sod1* (superoxide dismutase 1), *sod2*, *cat* (catalase), and *gpx1* (glutathione peroxidase 1) following NP uptake. In zebrafish, molecular oxygen (O_2_) acquires a single electron through the activity of NADPH oxidases (NOX) or via electron leakage from the mitochondrial electron transport chain (ETC), producing superoxide radicals (O₂**˙**⁻)^44,45^.These radicals are highly reactive and can cause cumulative oxidative damage, leading to cellular dysfunction and potentially death in zebrafish^46,47^. Naturally, to mitigate this, zebrafish possess endogenous antioxidant enzymes that neutralize reactive species. Superoxide dismutases (SODs)-notably *sod1* and *sod2*-constitute the first line of defense and possess crucial roles in cellular protection from oxidative stress by catalyzing the dismutation of two superoxide radicals into hydrogen peroxide (H_2_O_2_) and oxygen (O_2_)^48,49^. Although H_2_O_2_also serves as a signaling molecule, it remains potentially harmful because it can still promote oxidative damage despite being less reactive than superoxide radicals^50,51^. Accordingly, zebrafish further break down H_2_O_2_ through enzymes such as *cat* and *gpx1* into water (H_2_O) and, in the case of catalase, oxygen (O_2_)). Thus, increased expression of *sod* genes is considered to be indicative of elevated intracellular ROS. Furthermore, expression levels of *cat* and *gpx1* which act downstream in the antioxidant cascade, can neutralize ROS and reflect the capacity to convert hydrogen peroxide (H_2_O_2_) into water (H_2_O) and oxygen (O_2_), thereby helping maintain vital physiological functions.

**Fig. 4:**
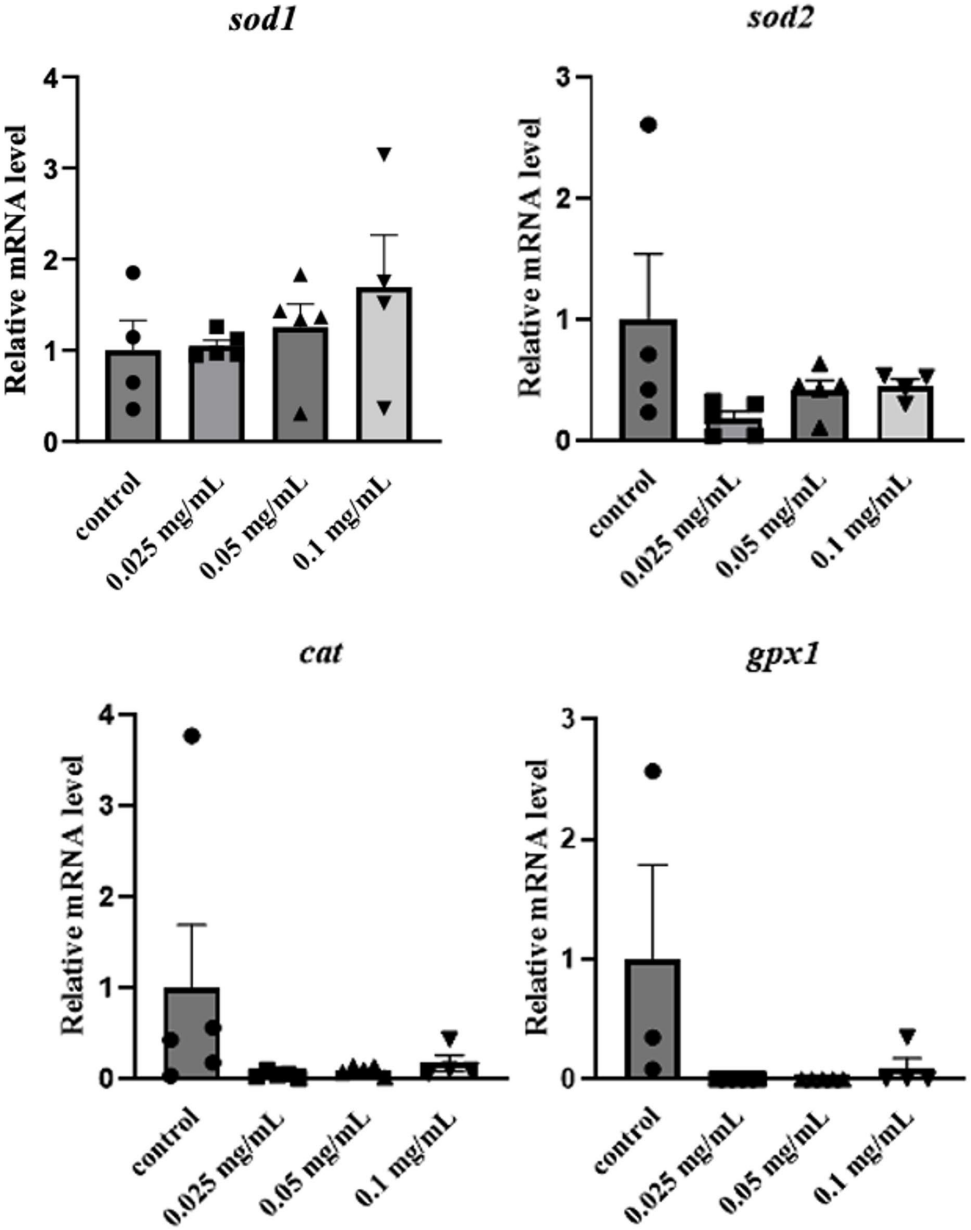
Relative expression levels of the *sod1*, *sod2*, *cat*, and *gpx1* genes of zebrafish embryos/larvae at 72 hpf to the *rpl13a* reference gene after exposure to various concentrations MnO_2_ NPs of 0.025, 0.05, and 0.1 mg/mL compared to the control.

**Fig. 5:**
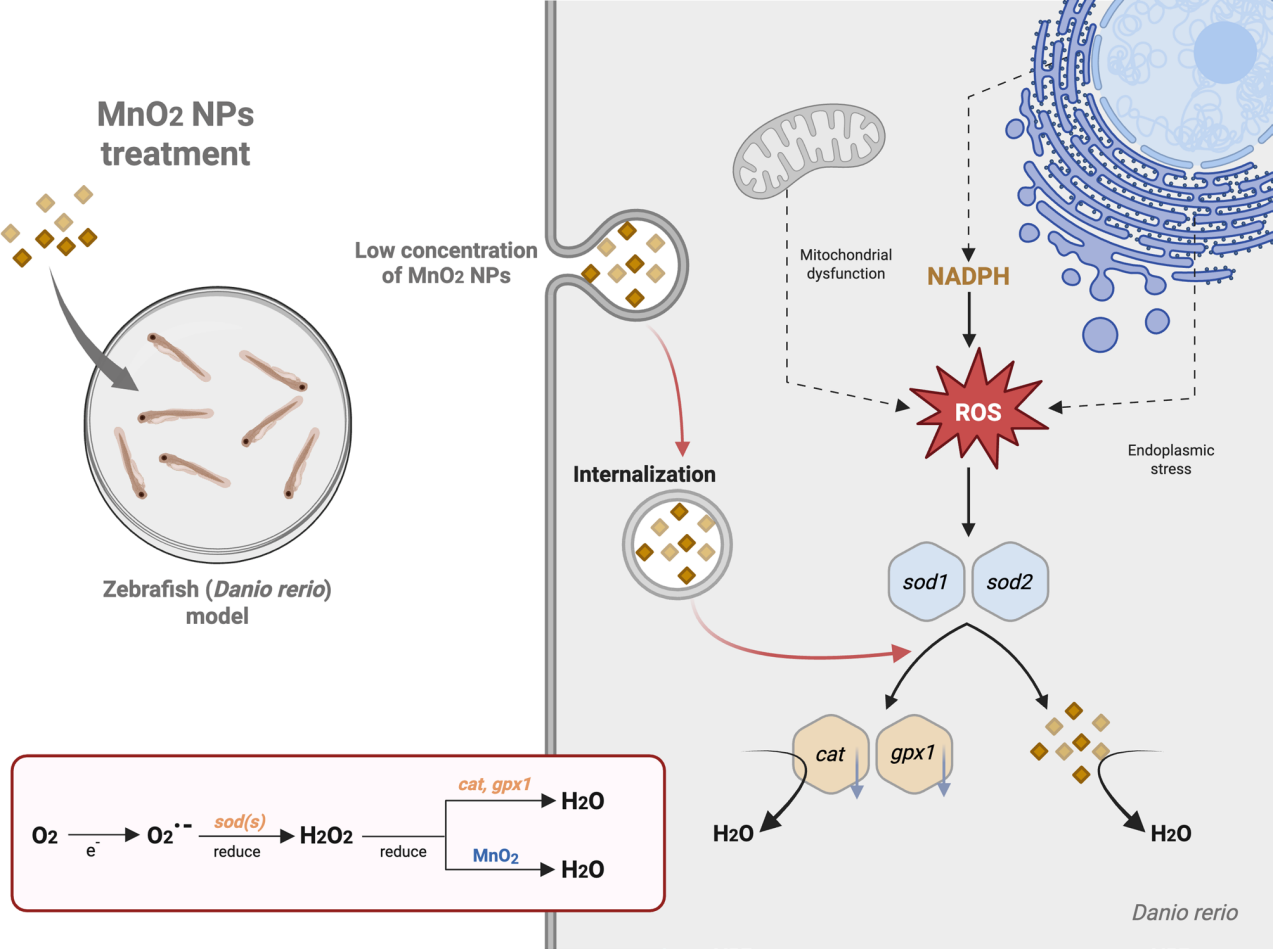
mRNA reaction pathway of marker genes *sod(s)*, *cat*, and *gpx1* for antioxidant regulation of zebrafish embryos after exposure to MnO_2_ NPs.

In this study, the qPCR analysis showed relative expression levels of *sod1*, *sod2*, *cat*, and *gpx1* genes of zebrafish embryos/larvae at 72 hpf with exposure to concentrations of 0.025, 0.05, and 0.1 mg/mL MnO_2_ NPs. According to the result in Fig. 4, gene expressions of zebrafish embryos/larvae with induced oxidative stress had an increasing trend in relative changes of *sod1* and *sod2*. Levels of both *sod1* and *sod2* were in line with MnO_2_ NP concentrations. As higher concentrations of MnO_2_ NPs were added, the superoxide concentration in zebrafish also increased. However, relative levels of *cat* and *gpx* remained unchanged. Figure 5 describes that *sod1* and *sod2* reduced the generated superoxide to hydrogen peroxide, and there are two pathways to further reduce it to water. One pathway involves the enzymes catalase (*cat*) and glutathione peroxidase (*gpx1*), which facilitate the direct reduction of hydrogen peroxide to water. The other pathway utilizes MnO_2_ NPs to catalyze the reduction of hydrogen peroxide to water. Both pathways effectively reduce hydrogen peroxide, contributing to the maintenance of cellular homeostasis. The contribution of MnO_2_ NPs in mimicking *cat* and *gpx1* enzymes aims to facilitate the reduction of hydrogen peroxide into water, thereby preserving the vital characteristics necessary for the fish’s survival. This mechanism is discussed under conditions where material concentrations are insufficient to cause fish death. These findings highlight the essential role of MnO_2_ NPs in mitigating ROS toxicity, with potential implications for studying valuable insights of MnO_2_ NPs in biomedical and toxicological research, and also in environmental and pathological oxidative stressors.

## Discussion

The potential toxicity of antioxidant-modified NPs must be rigorously evaluated prior to any pharmaceutical application, particularly when these nanomaterials are intended for drug delivery, therapeutics, or neuroprotection^52^. This is essential for both human health and environmental safety as the pharmaceutical sector has rapidly expanded NP use across biomedical (imaging, diagnostics, and therapeutics) and cosmetic applications^53,54^. Zebrafish (*D. rerio*) are widely employed for NP toxicity assessments to optimize efficacy and safety, to screen NP formulations and designs, and to assess biodistribution/bioaccumulation across organ systems (absorption, circulation, metabolism, and excretion)^55^. Having ~ 70% genetic similarity to humans and rapid development under low-maintenance laboratory conditions, zebrafish provide a robust vertebrate model for nanotoxicology^56–58^. Their optical transparency permits real-time, in vivo visualization of NP-tissue interactions and enables high-throughput screening, as many NPs passively diffuse into developing tissues^58,59^.

A central toxicological mechanism of NP exposure is oxidative stress, which serves as a sensitive, non-invasive biomarker of systemic dysfunction that correlates with behavioral and physiological abnormalities. Reported toxicity phenotypes include craniofacial malformations, pericardial edema, and spinal curvature^52^. NP-induced oxidative stress arises from an imbalance between ROS generation and endogenous antioxidant defense systems. ROS include superoxide (O₂•⁻), hydrogen peroxide (H₂O₂), and hydroxyl radicals (OH•⁻), which have physiological signaling roles but become harmful when overproduced^60^. For example, mitochondrial ROS accumulation can disrupt the electron transport chain (ETC), depress ATP production, and activate proapoptotic signaling pathways, ultimately perturbing cellular homeostasis and driving DNA damage and other pathologies^61^. A key measurable consequence of NP exposure is a weakening of the cellular defense system-i.e., disruption of the redox balance-reflected in activities of antioxidant enzymes such as *SOD*s, *cat*, and *gpx*^62^. Elevated ROS accumulation typically manifests as an early surge in SOD activity, the first line of antioxidant defense. Normally, under physiological conditions, cells maintain a natural antioxidant system to restrain ROS, thereby preventing excessive oxidative stress and averting apoptosis. The SOD-catalyzed reaction produces hydrogen peroxide (H_2_O_2_) and molecular oxygen (O_2_), as shown in equation (I)^63^. H_2_O_2_, which can still provoke oxidative stress, triggers subsequent upregulation of downstream enzymes. The peroxisomal enzyme catalase (*cat*) provides robust protection by decomposing H_2_O_2_, while glutathione peroxidase (*gpx1*), in conjunction with glutathione, reduces peroxidative species in both mitochondrial and cytosolic compartments, converting them to water and oxygen as summarized in equation (II). Expression levels of *sod1*, *sod2*, *cat*, and *gpx1* thus serve as informative readouts of ROS production, redox homeostasis, and the risk of cellular damage.

Superoxide dismutase (SOD) reduction mechanism:

1 $$2{O_2}^{ \cdot - } + 2{H^ + } \to {O_2} + {H_2}{O_2}$$

Catalase (*cat*) and glutathione peroxidase (*gpx1*) reduction mechanism:

2 $$2{H_2}{O_2} + 2{O_2} \to 2{H_2}O + {O_2}$$

Toxicity studies in zebrafish provide valuable insights into long-term risks associated with NP accumulation and help define safe concentration ranges. NPs can enter cells via active, receptor-mediated pathways or through passive processes such as fluid-phase endocytosis^64^. This study employed MnO_2_NPs, a biodegradable nanomaterial suitable for in vivo use without long-term toxicity concerns^65,66^. MnO_2_ NPs are inorganic, simple to prepare, inexpensive, and environmentally friendly, and exhibit high bioavailability. Manganese itself is a transition metal with multiple redox valences. Cellular studies showed that MnOₓ NPs possess enzyme-like activities-including oxidase, peroxidase, SOD-like, catalase, and glutathione peroxidase functions. More specifically, MnO_2_ NPs display high efficiency in generating O_2_ during H_2_O_2_’s decomposition. This performance is partly due to their ability to degrade and release water-soluble Mn²⁺ in the presence of H_2_O_2_ under acidic conditions. Biologically, Mn²⁺ ions act as cofactors for a wide range of enzymes, particularly those that detoxify superoxide radicals in the organism. Mn²⁺ can also be rapidly cleared by the kidneys under physiological conditions, thereby conferring low systemic toxicity.

Although many reviews have addressed the effects of metal and metal-oxide NPs in zebrafish, manganese dioxide NPs are seldom discussed, despite the excellent biosafety and degradability of MnO₂-based nanosystems^12^. Batir-Marin et al. examined oxidative-stress effects of metal-oxide NPs in zebrafish, including zinc oxide (ZnO NPs), titanium dioxide (TiO_2_ NPs) and iron oxide (Fe_2_O_3_ NPs), but there are no reports on the effects of MnO_2_NPs^52^. Mutalik et al. described nanomaterial toxicity studies of metal, metal oxide, semiconductor, and mixed-metal NPs; however the assessment did not include MnO_2_NPs^9^. Similarly, reviews on zebrafish neurotoxicity by Zhao et al., Haque & Ward, and d’Amora et al. surveyed numerous metal and non-metal NPs but did not report neurotoxicity attributable to MnO_2_NPs^54,56,58^. Given that MnO_2_NPs are among the more-stable nanomaterials with an immunomodulatory capacity and have broad applications-including cancer theranostics (photodynamic therapy, chemodynamic therapy, and immunotherapy), drug delivery, antimicrobial activity, cell capture, biocatalysis, biosensing, and tissue engineering-rigorous toxicity evaluations are essential to ensure non-toxic operating windows for biomedical use^67^..

MnO_2_ NPs have the ability to act as ROS scavengers due to their unique nature as a multienzyme that can mimic antioxidant enzyme activities such as by SODs and catalase. These features make MnO_2_ NPs strong candidates within the ROS-scavenger class and promising therapeutic alternatives for treating oxidative-stress-related conditions, as they degrade into Mn²⁺ ions and H_2_O molecules and do not accumulate in organs or tissues^67^. The form, size, polydispersity, and surface chemistry of MnO_2_ NPs are crucial things that can also influence their therapeutic effects. In this study, MnO_2_ NPs were synthesized at room temperature using a one-pot method by directly mixing the strong oxidant potassium permanganate (KMnO_4_) with the cationic polymer poly(allylamine hydrochloride) (PAH), spontaneously yielding a dark-brown colloidal suspension of MnO_2_NPs^68^. PAH acts both as a reductant of KMnO_4_ to MnO_2_and as a polyelectrolyte stabilizer, providing an electrostatic protective layer around the NPs^69,70^. The one-pot synthesize approach is noted for its simplicity, rapid and high-yield production, and cost efficiency^71–73^. Its single-vessel configuration minimizes waste, time, and byproducts, and typically requires no additional reagents or protective surfactants to ensure colloidal stability^74,75^..

Studies concluded that larger-sized NPs can minimize ROS production in zebrafish^52^. Small NPs such as those reported for TiO_2_NPs with a size of < 20 nm^76,77^AgNPs with a size of 10 nm^78^, AuNPs with a size of < 20 nm^79^, and CuNPs with a size of ≤ 20 nm^80^, could trigger more ROS production rather than larger-sized NPs. In this study, MnO_2_NPs as antioxidant-modified NPs had an average size of about 50 nm, so that their exposure to zebrafish did not cause an increase in ROS, thus increasing their bioavailability through prolonged oxidative stress and apoptosis^81,82^. In addition, surface coating of MnO_2_NPs in this study by PAH not only functioned as a stabilizer but also reduced ROS production, because it is able to protect NPs from aggregation and limits their internalization into cells, thereby improving their biocompatibility in the biological environment, according to previous studies^83,84^. PAH also provides a positive charge to the resulting MnO_2_NPs, which has a good impact on promoting NP adhesion onto an embryo’s surface^63^. Finally, the use of low NP doses in this study further supported low toxicity, as precise dosing can reduce cytotoxicity while improving drug-delivery efficiency^52^..

Establishing safe dosing windows for therapeutic NPs is essential to limit off-target effects. Work with metal NPs and polymeric carriers in zebrafish indicated that careful dose control can lower cytotoxicity while improving delivery efficiency^65^. These results underscore the complexity of NP-mediated delivery, where nanocarrier-bioactive compound interactions can shift pharmacodynamics and toxicity profiles. To resolve such variability, comprehensive pharmacokinetic studies are needed to define how NP formulations affect the absorption, distribution, and metabolism of therapeutics. Clarifying these mechanisms is critical to balance therapeutic benefits against unforeseen toxicological risks. Equally important is characterizing how specific NPs engage with cells and tissues to assess safety under clinical and environmental exposures. Looking ahead, priorities include chronic and multigenerational exposure studies and the use of omics technologies (transcriptomics, proteomics, and metabolomics) to deepen a mechanistic understanding of NP-biological system interactions^65^.

## Conclusion

The collective findings indicated that serial concentrations of MnO_2_ NPs (0.025, 0.05, and 0.1 mg/mL) did not exert overt toxic effects on zebrafish development. Instead, these concentrations appeared to support normal developmental processes by mitigating excessive oxidative stress. Anatomical assessments showed that embryos exposed to these low MnO_2_ NP concentrations progressed normally up to 72 hpf, with no delays in the formation of the eyes, brain regions, notochord, somites, fins, or other structures, and no detectable malformations. At the highest tested MnO_2_ NP concentration (0.1 mg/mL), embryos that survived largely exhibited normal development; however, mortality reached ~ 28% by 72 hpf. Although toxicity escalates with increasing doses and longer exposure times, developmental consequences remained subtle and below lethal thresholds. The antioxidant-response profile indicated that zebrafish embryos initiated a protective program marked by *sod1* level upregulation. In parallel, *cat* and *gpx1* downregulation was supported by MnO_2_ NPs’ catalase- and glutathione peroxidase-mimetic activities that detoxified hydrogen peroxide and facilitated its conversion to water. Taken together, these results imply that at low doses, MnO_2_ NPs are relatively benign or even beneficial to embryos, and they did not significantly derail embryogenesis or induce the embryos’ antioxidant defense system. In essence, low-level MnO_2_ NP exposure appeared to be below the toxicity threshold for zebrafish early-life stages, allowing normal anatomical development while mildly activating defense pathways.

## Data Availability

All data generated or analyzed during this study are included in this article.
